# Sir Thomas Watson, M.D., on Animal Vaccination

**Published:** 1880-01

**Authors:** 


Sir Thomas Watson, M.D., on Animal Vaccination. On the subject of vaccination with bovine virus, the eminent London physician, Sir Thomas Watson, writes to the *Pall Mall Gazette:* "Several letters have lately appeared in the *Times* newspaper, respecting what is called animal vaccination. In one of the numbers of the *Nineteenth Century* vaccination on and from the calf was earnestly advocated by me, as carrying with it the potential extinction of the only valid objection that can be alleged against vaccination in general; and justifying, therefore, the needful compulsion of vaccination by force of law. The anti-vaccination party have attempted to enroll me among the writers who have adopted their views. I ask leave, therefore, to inform you and your readers that my sole object has been, and is, to prove by vaccination, properly and universally effected, the hideous, disfiguring, dangerous, and in a majority of cases, fatal distemper, small-pox, may with certainty be extirpated from this country—I might say from this world."—*Medical and Surgical Reporter.*

